# Hybrid forward-selection method-based water-quality estimation via combining Landsat TM, ETM+, and OLI/TIRS images and ancillary environmental data

**DOI:** 10.1371/journal.pone.0201255

**Published:** 2018-07-30

**Authors:** Min-Cheng Tu, Patricia Smith, Anthony M. Filippi

**Affiliations:** 1 Department of Civil and Environmental Engineering, Villanova University, Villanova, Pennsylvania, United States of America; 2 Villanova Urban Stormwater Partnership (VUSP), Villanova University, Villanova, Pennsylvania, United States of America; 3 Department of Biological and Agricultural Engineering, Texas A&M University, College Station, Texas, United States of America; 4 Department of Geography, Texas A&M University, College Station, Texas, United States of America; 5 Center for Geospatial Science, Applications and Technology (GEOSAT), Texas A&M University, College Station, Texas, United States of America; Zhongnan University of Economics and Law, CHINA

## Abstract

A simple approach to enable water-management agencies employing free data to create a single set of water quality predictive equations with satisfactory accuracy is proposed. Multiple regression-derived equations based on surface reflectance, band ratios, and environmental factors as predictor variables for concentrations of Total Suspended Solids (TSS) and Total Nitrogen (TN) were derived using a hybrid forward-selection method that considers both p-value and Variance Inflation Factor (VIF) in the forward-selection process. Landsat TM, ETM+, and OLI/TIRS images were jointly utilized with environmental factors, such as wind speed and water surface temperature, to derive the single set of equations. Through splitting data into calibration and validation groups, the coefficients of determination are 0.73 for TSS calibration and 0.70 for TSS validation, respectively. The coefficients of determination for TN calibration and validation are 0.64 and 0.37, respectively. Among all chosen predictor variables, ratio of reflectance of visible red (Band 3 for Landsat TM and ETM+, or Band 4 for Landsat OLI/TIRS) to visible blue (Band 1 for Landsat TM and ETM+, or Band 2 for Landsat OLI/TIRS) has a strong influence on the predictive power for TSS retrieval. Environmental factors including wind speed, remote sensing-derived water surface temperature, and time difference (in days) between the image acquisition and water sampling were found to be important in water-quality quantity estimation. The hybrid forward-selection method consistently yielded higher validation accuracy than that of the conventional forward-selection approach.

## 1. Introduction

Continuous monitoring of water quality is essential for the health and welfare of the people and ecosystems reliant upon them. Urbanization, agriculture, and other anthropogenic factors can alter water quality [1], and waiting to remediate until a change is clearly visible can be much more costly than early prevention. Despite this, the cost of adequate temporal and spatial physical measurements can potentially be prohibitive [2]. For example, the United States Geological Survey (USGS) regularly monitors water quality in Lady Bird Lake in Austin, Texas, USA; however, the frequency is only approximately twice per year at a single point near the outlet over the past decade [3]. Additionally, *in situ* measurements from year to year do not occur in the same months. As a result, it is difficult to distinguish whether a change in the water quality measured at a point is truly a long-term change or the result of a seasonal difference or recent event (e.g., a large precipitation event) [4]. Additionally, it is impossible to evaluate the spatial variation in water quality from single-point measurements.

In recent decades, remote sensing has provided an alternative method for monitoring water quality in a spatially synoptic manner at a lower cost compared with extensive *in situ* measurement. Each water-column constituent exhibits a specific spectral response that can be observed by satellite- and aircraft-mounted remote sensors [5]. Suspended sediment usually exhibits strong backscattering of incident light [5], where the actual color depends on the terrestrial origin [6]. CDOM generally exhibits an exponential reduction in absorption with increasing wavelength; CDOM spectral absorption curves typically entail strong absorption features in the ultraviolet to blue wavelength region (280–400 nm), with dramatic decreases to near zero in the red and near infrared portions of the spectrum [7]. Chlorophyll a (e.g., in algae-laden waters) entails strong absorption in the blue and red portions of the spectrum, as well as a reflectance maximum around 550 nm (i.e., a green peak) [8]”.

For a particular wavelength, *λ*, the spectral radiance from the water observed vertically, known as the upwelling radiance, *L*_*u*_, is given by

$$L_{u}(\lambda )=L_{w}(\lambda )+\Omega L_{s}(\lambda )$$ (1)

where *L*_*w*_ is the radiance reflected/backscattered by the water column, in-water constituents, and the bottom if the water column is optically shallow; *L*_*s*_ is the skylight radiance; and Ω is the ratio of radiance directly reflected by the water surface to *L*_*s*_ [9]. Note that the radiance observed by a satellite is composed of *L*_*u*_, plus atmospheric interference; therefore, it requires atmospheric correction (discussed below). *L*_*w*_, *L*_*s*_, and Ω are influenced by a variety of factors. If the water column is sufficiently deep, bottom reflectance may be ignored, and *L*_*w*_ can be assumed to be a measure of the effects of water-column constituents alone. Atmospheric conditions (e.g., clear, cloudy, overcast) affect both Ω and *L*_*s*_, whereas Ω can be further affected by wind speed in the form of surface ripples (e.g., temporary sun glint) [9]. Wind speed has also been found to have some influence on water clarity [10].

Because of their higher capability to penetrate the water column, visible bands have conventionally been used to estimate water quality [5]. In addition, infrared bands have also shown significance in determining water-quality quantities in some studies [11, 12]. However, only near infrared wavelengths were used in these studies. Thermal infrared bands have not extensively been used in water-quality estimation.

Site-specific predictive models can be created to relate a number of band radiance measurements or derived reflectance values [5] to the water-quality quantity of interest by fitting the model to *in situ* water-quality measurements. Multiple regression analysis and artificial neural networks (ANNs) constitute two methods that are frequently used to generate such predictive models [5, 12, 13, 14].

In academia, satellite remote-sensing images have been increasingly available for water-quality determination. However, the popularity of this approach has not been extended to decision making by management agencies in general [15]. According to Schaeffer et al. [15], the reasons for this phenomenon include cost, product accuracy, data continuity, and programmatic support.

Cost is always a major constraint, as many water-management agencies have limited budgets [15]. Even though there are many free remote-sensing data sets available, such as the multispectral satellite images available from the Landsat program (e.g., Landsat Thematic Mapper (TM), Enhanced Thematic Mapper Plus (ETM+), and Operational Land Imager (OLI)/ Thermal Infrared Sensor (TIRS)) [16], MODIS [17], SeaWiFS [18], etc., terrestrial pond/lake applications are predominately limited to moderate spatial-resolution images from the Landsat program due to its relatively finer spatial resolution. Another aspect of the cost constraint is the cost to collect field water-sampling data, as the creation of empirical predictive models necessitates *in situ* water-quality data. Sometimes, due to cost, logistical, and other constraints, a water-management agency can only resort to free water-quality data, such as those made available by the USGS. The downside, as noted above, is that spatio-temporal sampling density/data availability may be low. This drawback seriously limits the ability of a water-management agency to utilize free Landsat program data, for example, as the basis of a water-quality monitoring program since the satellite images and corresponding *in situ* measurements must be acquired in a temporally proximal manner [19]. Furthermore, water-quality variables of interest may not even be measured, given the complexity or cost of the measuring techniques needed, making regular/automatic sampling difficult.

As a result of these issues, water-management agencies that resort to using only free data resources often have access to a limited number of useable satellite images for water-quality monitoring. Such a scenario often leads to the use of a single predictive model to determine water-quality information from satellite images. Nevertheless, many studies divide their analyses by season [20, 21] due to systemic seasonal differences in factors such as concentrations of color-producing substances (including phytoplankton), atmospheric disturbances [21], and solar zenith angle [22]. Some studies have shown that the predictive power of equations created without distinguishing by season is lower than it would otherwise be [23, 24].

Since the derived predictive equation is seasonally affected by the environment, a few studies have incorporated the influencing factors into predictive equation generation. One example is with the estimation of *chlorophyll-a* concentration. It is known that phytoplankton growth is statistically significantly dependent on water temperature [25, 26]. Incorporating water temperature (derived from the satellite remote-sensor thermal band) in development of predictive equations has proven to be helpful in determining *chlorophyll-a* concentration [27]. However, this approach has not been investigated extensively. In this study, we consider additional environmental factors based on energy fluxes between a waterbody and the atmosphere. We posit that including these environmental factors in predictive equations not only increases prediction accuracy, but also facilitates the usage of a single set of predictive equations throughout different seasons. The direct benefit is that one can pool all observation data in creating equations, thus resulting in higher predictive power.

Programmatic support is also important to water-management agencies, according to Schaeffer et al. [15]. In most cases, local universities should be sufficient in providing support to water-management agencies. However, we posit that the methodology adopted for generating predictive models should entail model construction in a step-wise manner, such that most people with basic training could implement itwithout much difficulty. For this reason, in choosing a methodology to be implemented by water-management agencies, simple and well-understood methods such as multiple regression should be weighed against more complex methods, such as ANNs.

Product accuracy is another major concern expressed by the water-management agencies [15]. Even though water-management agencies could utilize predictive models from peer-reviewed journals, such models may not yield high-accuracy estimates in a given application. Multiple regression analysis has been employed in many studies for its ease of application. However, for applications using this method, overfitting from multicollinearity can be a serious concern. Multicollinearity means that some of the explanatory variables in the multiple regression model are dependent on one another. The direct result of multicollinearity is that the standard error of coefficients of explanatory variables is inflated, which means that coefficients of the derived model are not reliable. Unfortunately, many past studies neither discuss the issue of multicollinearity, nor provide results of validation of the derived regression models [4, 5, 11, 19, 28, 29, 30, 31]. A common way to identify multicollinearity of a model is through the use of indicators such as Akaike’s Information Criteria [32], Mallow’s Cp [33], PRESS [34], etc. However, such indicators apply to the whole model so all possible subsets of explanatory variables must be examined, and this approach becomes difficult when the number of variables increases [35], even with modern computing power.

Other popular methods to identify multicollinearity include the deployment of principal component analysis (PCA) or structural equation modeling (SEM) [35]. PCA creates orthogonal principal components, which are linear combination of variables, and a regression model can be created based on the orthogonal components in order to eliminate multicollinearity completely. However, some studies show that this methodology can result in a loss of explanatory power. Additionally, the main limitation of the PCA approach is that physical interpretations of the principal components are required. On the other hand, SEM accepts the existence of collinearity among explanatory variables and hypothesizes that a model exists among variables. Then all possible combinations of causal links among variables are tested against the hypothesized model. Since SEM is not an exploratory technique, SEM is prone to inferential errors made during development and selection of the hypothetical models [35].

We use the variation inflation factor (VIF) in step-wise variable selection, which is based on p-value, to minimize multicollinearity. Unlike other indicators described above, VIF is calculated for each predictor variable. VIF has been used in the field of remote sensing on a limited basis to check multicollinearity of results [36, 37]. Dubovyk et al. [38] used VIF to choose variables to enter into a logistic regression model. VIF has not previously been incorporated along with established variable-selection methods (e.g., forward step-wise selection) to derive predictive equations for water-quality quantities. Details regarding VIF computation and the methodology to include VIF in equation derivation is discussed below in the Methodology section.

Although the Landsat program entails a few limitations, such as the inflexible satellite overpass schedule and the relatively lower sensitivity of sensors prior to Landsat 8, the Landsat program constitutes a truly ideal free data-source candidate for water-management agencies, given the characteristics of the various Landsat sensors, as well its long-term data continuity. The Landsat program has maintained the longest uninterrupted satellite observation record of Earth from its beginning in 1970s, employing several sensors over time including MSS, TM, ETM+, and OLI/TIRS (Landsat 8), with improving sensor sensitivities. Only a few water-quality studies have taken advantage of combining TM, ETM+, and OLI/TIRS datasets [39, 40, 41] even though these sensors have been shown to be compatible, as shown in Table 1 [41, 42, 43]. Note Table 1 shows only comparable bands among Landsat TM, ETM+, and OLI/TIRS sensors.

**Table 1:** Band attributes of Landsat TM and ETM+ and OLI/TIRS sensors [41, 42, 43].

		**Band 1**	**Band 2**	**Band 3**	**Band 4**	**Band 5**	**Band 6**	**Band 7**	**Band 8**
**TM**	**Wavelength (μm)**	0.45–0.52	0.52–0.60	0.63–0.69	0.76–0.90	1.55–1.75	10.40–12.50	2.08–2.35	n/a
	**Sensor spatial resolution (m)**	30	30	30	30	30	60	30	n/a
**ETM+**	**Wavelength (μm)**	0.45–0.52	0.52–0.60	0.63–0.69	0.77–0.90	1.55–1.75	10.40–12.50	2.09–2.35	0.52–0.90
	**Sensor spatial resolution (m)**	30	30	30	30	30	60	30	15
**OLI/TIRS**	**Wavelength (μm)**	**Band 2**	**Band 3**	**Band 4**	**Band 5**	**Band 6**	**Band 10**	**Band 7**	**Band 8**
		0.45–0.51	0.53–0.59	0.64–0.67	0.85–0.88	1.57–1.65	10.60–11.19	2.11–2.29	0.50–0.68
	**Sensor spatial resolution (m)**	30	30	30	30	30	100	30	15

Because band numbering is different in OLI/TIRS, in this study, band numbers will be based on TM/ETM+. For example, if Band 3 is noted, it means Band 3 for TM and ETM+, but Band 4 for OLI/TIRS.

Based on the gaps in the research literature illustrated above, the objectives of this study were:

1. Incorporate environmental factors (such as temperature, wind speed, etc.) into a single set of predictive equations for remote-sensing water-quality measure estimation; and
2. Increase model predictive power for a limnological water-quality quantity-estimation application by considering the effect of multicollinearity in established model-creation methodologies such as forward step-wise selection.

The goal of this study is to address all four concerns of utilizing satellite data in decision making by water-management agencies—i.e., cost, product accuracy, data continuity, and programmatic support. This study provides water-management agencies with a simple, easy-to-follow methodology for utilizing free observation data (from Landsat program, USGS, etc.) in order to address cost and programmatic-support issues for water-quality monitoring. The Landsat program guarantees long-term data continuity. The proposed methodology provides a single set of predictive equations; accuracy is maintained because all available data are consolidated for the creation of a single model. Also, consideration of multicollinearity increases the likelihood for acceptable estimation accuracy of the derived model in future water-quality quantity retrieval applications.

## 2. Materials and methods

### 2.1 Study area

**Fig 1:**
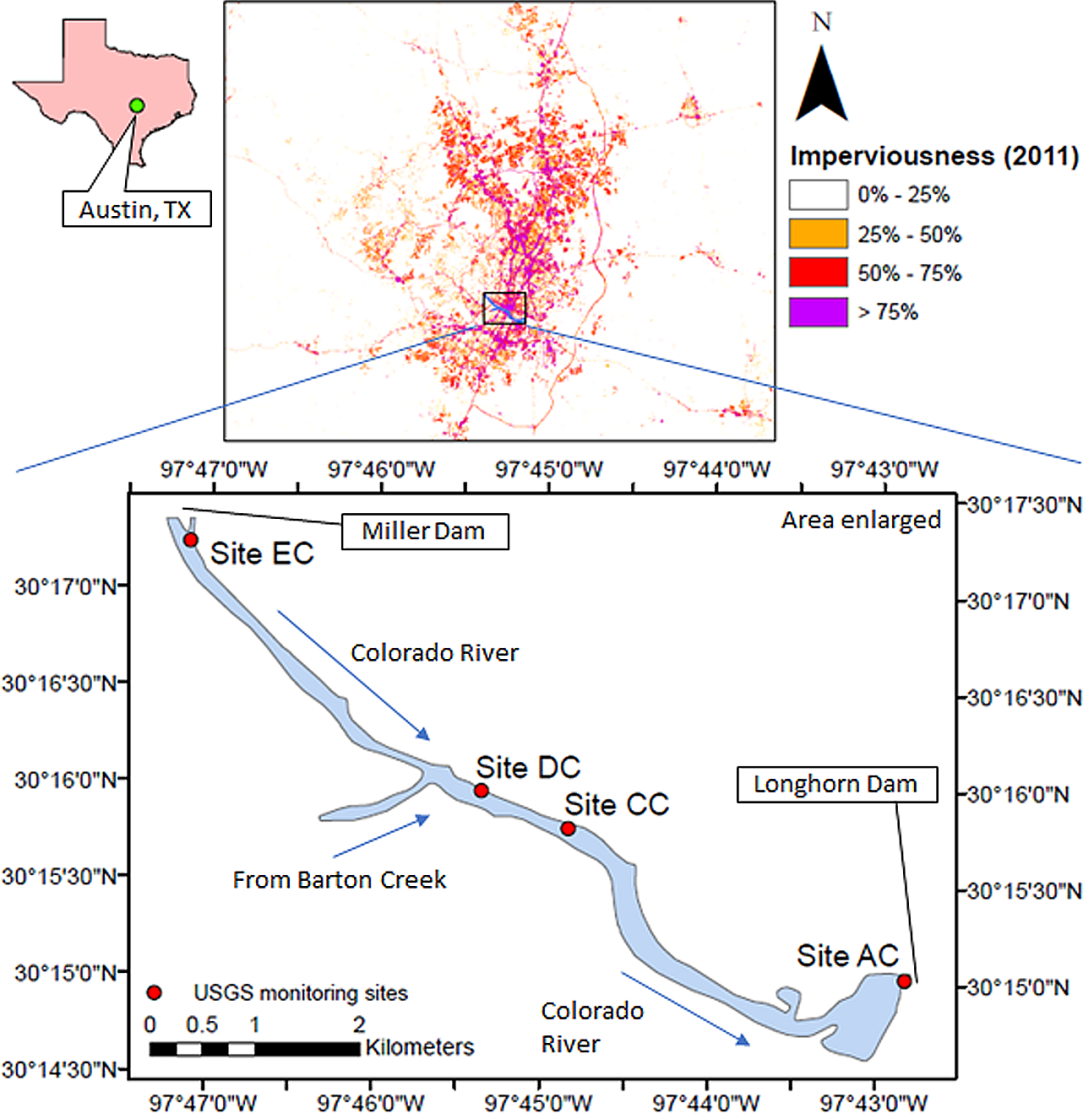
Locations of water-quality sampling stations (i.e., Sites AC, CC, DC, and EC) on Lady Bird Lake.

The population of City of Austin, Texas, USA has increased dramatically in recent decades, from 346,000 in 1980 to 968,000 in 2018 [44]. With significant population growth comes an increase in impervious area, higher runoff and lower water quality in local water bodies. Lady Bird Lake (formerly Town Lake), situated near the city center, provides an opportunity to remotely monitor water quality in an urban watershed (Fig 1). The lake, formed by damming the Colorado River, is maintained at an approximately constant level by the pass-through Longhorn Dam [45]. The surface area is ~1.74 square kilometers with a capacity of 9,051,000 cubic meters. The mean depth is 6 meters, with a maximum depth over 11.7 meters [46].

The USGS maintains a number of water-quality sampling stations on Lady Bird Lake, but only four of them, EC, DC, CC and AC (Fig 1), monitor the water-quality constituents of interest in this study within the time frame of available satellite images (i.e., 1983–2015) [3]. Table 2 provides basic information for these four sampling stations, including summary statistics for these water-quality quantities of interest—total suspended solids (TSS) and total nitrogen (TN)—derived from water-quality samples collected at a depth of 1 m. Secchi disc transparency, a pseudo-measure of turbidity, was measured in four locations when the samples of Table 2 were taken (Table 3). Secchi disc depths were much shallower than the average bottom depth of the lake (6 m); thus, bottom reflection is not observable from above the air-water interface for these cases. Therefore, contribution of bottom reflectance to the water-leaving radiance (Eq 1) can be ignored.

**Table 2:** Summary statistics from *in situ* USGS water-quality stations in Lady Bird Lake, Texas, USA, over the time period 1983–2015.

USGS Water Quality Stations and Site Codes	Water-Quality Measures and USGS Parameter Code					
	TSS (mg/L) 00530^a^			TN (mg/L) 00600^a^		
	# of Samples	Mean	Std. Dev.	# of Samples	Mean	Std. Dev.
**EC 301712097470701**^b^	7	4.57	4.24	11	0.58	0.22
**DC 301558097452201**^b^	8	5.75	5.39	8	0.71	0.36
**CC 301546097445101**^b^	4	9.50	5.26	6	0.53	0.14
**AC 301500097424801**^b^	9	8.44	10.35	13	0.71	0.25
**All**	28	6.86	7.06	38	0.64	0.26

^a^ Water-quality quantity code as assigned by USGS

^b^ USGS station number

**Table 3:** Secchi disc transparency measurements for *in situ* USGS water-quality stations in Lady Bird Lake, Texas, USA, over the time period 1983–2015.

Site Code	# of measurements	Mean (m)	Std. Dev. (m)
**EC**	11	2.22	0.86
**DC**	10	1.68	0.77
**CC**	8	1.23	0.62
**AC**	15	1.27	0.60

### 2.2. Selection of satellite images

Selection of Landsat TM, ETM+, and OLI + TIRS images [47] was based on several criteria. Images selected were cloud-free and were acquired within seven days of *in situ* water-quality measurements in Lady Bird Lake [12, 20]. In order to minimize the effects of spatio-temporally-close rainfall events, only images that occurred when daily precipitation depths observed between the dates of the selected images and their associated water-sampling dates were less than 1.25 cm (0.5 inch) were selected (Table 4). This threshold rainfall depth is chosen based on the initial abstraction rainfall depth for a watershed with a runoff curve number of 80, since most of the urbanized area around Lady Bird Lake is residential [48]. Residential districts with small lot sizes (1/4 to 1/8 acre) have a curve number ranging from 61 to 92, depending on the soil hydrologic group [49]. Rainfall depth below this threshold is considered to generate insignificant runoff, and thus should have no marked effect on water quality in the lake.

**Table 4:** Dates of Landsat TM and ETM+ satellite images utilized and respective corresponding water-quality samples.

Sensor Name	Image Date	Water-Quality Sampling Date
Landsat 4 TM	January 9, 1983	January 6, 1983
Landsat 5 TM	August 18, 1985	August 20, 1985
Landsat 5 TM	January 15, 1988	January 19, 1988
Landsat 5 TM	April 20, 1988	April 19, 1988
Landsat 5 TM	July 25, 1988	July 27, 1988
Landsat 5 TM	March 6, 1989	February 27, 1989
Landsat 5 TM	April 7, 1989	April 12, 1989
Landsat 5 TM	August 5, 1992	August 10, 1992
Landsat 5 TM	July 24, 1999	July 22, 1999
Landsat 5 TM	December 20, 2001^a^	December 16, 2001
Landsat 7 ETM+	April 22, 2009	April 18, 2009
Landsat 5 TM	June 4, 2010	June 3, 2010
Landsat 7 ETM+	May 14, 2011	May 13, 2011
Landsat 8 (OLI + TIRS)	May 14, 2014	May 14, 2014
Landsat 8 (OLI + TIRS)	March 14, 2015	March 10, 2015

^a^ Excluded from analysis due to issues with atmospheric correction.

### 2.3. Atmospheric correction

#### 2.3.1. Image Pre-processing

Surface reflectance values corrected for path radiance were derived using Fast Line-of-sight Atmospheric Analysis of Spectral Hypercube (FLAASH^®^) radiative transfer model [50, 51]. Remote-sensing reflectance from spectrally dark targets such as Lady Bird Lake is usually much lower than that from the surrounding urban areas [52]. With FLAASH, significant errors can occur when strong albedo contrasts exist among the materials in the scene [51]. To minimize this potential problem, a land mask was created and applied in order to exclude all surrounding land regions [53], leaving just the aquatic areas (i.e., Lady Bird Lake) for subsequent atmospheric-correction processing.

#### 2.3.2. Determination of FLAASH parameter values

Two of the parameters required by FLAASH are visibility and choice of atmospheric model. Visibility obtained from historical airport records [54] likely caused FLAASH to over-compensate in its correction of atmospheric effects and yield negative reflectance values probably because the highest reported visibility is limited at 6 miles (9.6 km) [55] and visibility higher than that is not discernable from airport records. Therefore, the 2-band (K-T) aerosol retrieval method [51] with “urban” setting was used to estimate visibility. Ideally, selection of an atmospheric model is based on one of the following options, presented in order from most preferred to least preferred: known standard column water vapor amount, expected surface air temperature, or tabulated seasonal-latitude combinations [51]. Although there are atmospheric water-content products available [56], they do not cover all dates of interest in this research. Surface temperatures have been continuously recorded and archived by Camp Mabry Austin City Airport and Austin Bergstrom International Airport every hour over the past 30 years [54]. Therefore, atmospheric models were selected based on the surface air temperature at the time when each satellite image was acquired (Table 5). The initially-selected December 20, 2001 image was excluded from subsequent processing because it yielded negative reflectance values after FLAASH atmospheric correction.

**Table 5:** Selection of FLAASH atmospheric model based on measured surface air temperature.

Image Date	Surface Air Temperature (°C)	Chosen Atmospheric Model	Suggested Temperature for Model (°C) [51]
January 9, 1983	11	Sub-Arctic Summer	14
August 18, 1985	33	Tropical	27
January 15, 1988	10	Sub-Arctic Summer	14
April 20, 1988	23	Mid-Latitude Summer	21
July 25, 1988	31	Tropical	27
March 6, 1989	2	Mid-Latitude Winter	-1
April 7, 1989	25	Tropical	27
August 5, 1992	30	Tropical	27
July 24, 1999	32	Tropical	27
December 20, 2001	11	Sub-Arctic Summer	14
April 22, 2009	31	Tropical	27
June 4, 2010	31	Tropical	27
May 14, 2011	23	Mid-Latitude Summer	21
May 14, 2014	21	Mid-Latitude Summer	21
March 14, 2015	22	Mid-Latitude Summer	21

### 2.3.3. Atmospheric correction for thermal bands

FLAASH should not be applied to thermal bands [51]; therefore, another atmospheric-correction method was applied to thermal bands. In particular, the single-band atmospheric-correction method described by Barsi et al. [57] was used. The methodology calculates atmospheric transmission and path radiance using MODTRAN [51], based on the atmospheric profiles generated by National Centers for Environmental Prediction (NCEP). Eq 2 provides the relationship between top-of-atmosphere radiance (L_TOA_), the target radiance of kinetic temperature T (L_T_), the path (upwelling) radiance (L_a_), and the sky (downwelling) radiance (L_d_):

$$L_{TOA}=\tau \varepsilon L_{T}+L_{a}+\tau (1-\varepsilon )L_{d}$$ (2)

In Eq 2, atmospheric transmission τ, path radiance *L*_*a*_, and sky radiance *L*_*d*_ were obtained from the on-line calculator based on the atmospheric correction method of Barsi et al. [57]. Since water is a near-perfect blackbody, emissivity (ε) was set as 1 in this study according to Haydon [58]. Emissivity and transmission are unitless, whereas radiance values are in units of W/m^2^·sr·μm.

The atmospheric profiles are only available after January 2000. For satellite images acquired prior to that, atmospheric profiles from “surrogate dates” in 2000 were used in this study. The surrogate date has nearly identical daily precipitation, temperature, and wind speed as the satellite image date. By choosing a surrogate date in such a manner, the atmospheric condition of the actual satellite image date and the surrogate date are expected to be similar. If more than two surrogate dates were found based on the above criteria for one satellite image, the one that is temporally closest to the date in the year in which a given the satellite image was acquired was chosen. Table 6 provides the list of the satellite image dates, the corresponding surrogate dates, and daily meteorological parameters for both of them.

**Table 6:** Comparison between image and surrogate dates in atmospheric profile determinations.

	Image date weather parameters				Surrogate date weather parameters		
Satellite Date	Daily rainfall (mm)	Daily Mean Temp (^o^C)	Daily mean wind speed (m/s)	Surrogate Date	Daily rainfall (mm)	Daily Mean Temp (^o^C)	Daily mean wind speed (m/s)
**Jan 9, 1983**	0	11	3.1	**Dec 20, 2000**	0	11	3.1
**Aug 18, 1985**	0	31	3.6	**Aug 28, 2000**	0	32	3.6
**Jan 15, 1988**	0	9	2.8	**Nov 13, 2000**	0	9	3.6
**Apr 20, 1988**	0	21	3.6	**Apr 22, 2000**	0	20	3.4
**Jul 25, 1988**	0	30	3.1	**Jul 26, 2000**	0	31	3.2
**Mar 6, 1989**	0	3	5.8	**Dec 27, 2000**	0	3	4.1
**Apr 7, 1989**	0	22	2.8	**May 14, 2000**	0	23	2.8
**Aug 5, 1992**	0	29	3.1	**Aug 20, 2000**	0	30	3
**Jul 24, 1999**	0	29	1.7	**Jul 24, 2000**	0	29	1.6

#### 2.3.4. Determining surface temperature from landsat thermal bands

Target temperature (i.e., water surface temperature) was derived after atmospheric correction according to equations provided in the Landsat Data User Manual [43]. For Landsat ETM+, the low-gain channel was used because it has a wider dynamic range and is not easily saturated [59]. For Landsat TIRS, only band 10 was used because data from band 11 have been contaminated by a stray-light effect, and a remedy has not yet been found [60]. Bands 10 and 11 here are band numbering from Landsat TIRS.

#### 2.3.5. Post-processing for atmospherically-corrected surface reflectance

Surface reflectance values at the water-quality stations were extracted from the FLAASH-corrected satellite images. Pixels located at the exact coordinates of the respective water-quality sampling stations are not necessarily the ideal pixels for which reflectance values should be extracted for analysis. The USGS water-quality stations are all positioned very close to the shore or land-related objects (such as bridges); thus, the pixel located at the exact coordinates of a given water-quality sampling station may contain land and/or land-related objects.

To minimize potential deleterious effects of such mixed pixels, the search range was expanded to 90 m (i.e., a search neighborhood comprised of 3 × 3 image pixels, centered on the pixel located at the station coordinates). The pixel within this zone with the lowest value in band 5 was chosen as the representative pixel, as it is the pixel to most likely contain only water [61]. If two pixels had the same band 5 values, the pixel closest to the coordinates of water-quality sampling location was selected.

## 3. Multiple regression analysis

Multiple regression equations were derived to predict constituent concentrations (TSS and TN, i.e. the dependent variables) from the predictor variables, such as band reflectance. The procedure for selection of predictor variables is delineated below.

The spectral bands and associated band ratios were all chosen as candidates for independent variables. Band ratios were included as independent variables in the regression analysis [12] because they are less apt to be influenced by lighting conditions [62].

Radiance data from the thermal bands (band 6 of Landsat TM and ETM+, and band 10 of Landsat TIRS) were converted to water surface temperature. As discussed earlier, water temperature has been found to be related to phytoplankton concentration [23, 24], and thus, related to water quality [63]. However, in this study, most of the satellite image dates differ by several days compared with the closest corresponding actual water-quality sampling date; thus, the water surface temperature derived from the satellite images does not represent the actual water temperature at the time of water sampling.

Eq 3 considers the net energy fluxes between a waterbody and the atmosphere [64]:

$$NET={SWR}_{net}-({LWR}_{net}+LHF+SHF)$$ (3)

where NET is the net energy flux, SWR_net_ indicates the net short-wave radiation energy flux (Eq 4), LWR_net_ indicates the net long-wave radiation flux (Eqs 5 and 6), LHF is the latent heat flux (Eq 7), and SHF is the sensible heat flux (Eq 8). These terms are calculated by the following equations [64]:

$${SWR}_{net}=(1-a){SWR}_{down}$$ (4)

$${LWR}_{net}\approx \varepsilon \sigma {T_{s}}^{4}(0.39-0.05{e_{a}}^{\frac{1}{2}})(1-0.51C^{2})+4\varepsilon \sigma {T_{s}}^{3}(T_{s}-T_{a})$$ (5)

$$C\approx 1.61(1-\frac{{SWR}_{down}}{{SWR}_{cs}}+0.0019n)$$ (6)

$$LHF=\rho L_{e}C_{e}U(Q_{s}-Q_{a})$$ (7)

$$SHF=\rho C_{p}C_{h}U(T_{s}-T_{a})$$ (8)

where *a* is the surface albedo (usually very low for water so SWR_net_ ≈ SWR_down_), ε is the surface emissivity, σ is the Stefan-Bolzman constant, T_s_ is the water surface temperature, T_a_ is the air temperature, e_a_ is the surface vapor pressure, C is the cloud cover index (Eq 6), SWR_cs_ is the clear-sky short wave radiation, n is the noon solar altitude, *ρ* is the density of air, L_e_ is the latent heat of evaporation, C_e_ is the turbulent exchange coefficient for latent heat, U is the wind speed, Q_s_ and Q_a_ are saturation specific humidity at the surface and at near-surface atmosphere, respectively, and C_h_ is the turbulent exchange coefficient for sensible heat.

Some of the variables in Eqs 4 to 8 are known or can be reasonably assumed as constants (*a*, ε, σ, *ρ*, L_e_, C_e_, and C_h_ [65]). The surface vapor pressure, e_a_, is dependent on water surface temperature [66]. Q_s_ and Q_a_ are both dependent on temperature as well [67]. The air temperature and noon solar altitude (T_a_, and n respectively) can be obtained from the historical observation record. The water surface temperature T_s_ is obtained from thermal band data. That leaves only one variable unknown, which is the clear-sky short wave radiation SWR_cs_. Calculating SWR_cs_ involves a complex procedure [68] so it is difficult to associate it with distinct environmental factor(s); thus, we did not consider it in evaluating heat flux in this study.

Assuming that the temperature change between the image date and the water-sampling date directly corresponds with the cumulative heat flux between the dates, the following variables are needed in order to account for the temperature change between the image-acquisition date and the water-sampling date [54]:

1. Time offset (in days) between the image date and the water-quality sampling date (positive offset means that the image date is later than the sampling date);
2. Water surface temperature (in K) derived from the thermal band;
3. Air temperature (in K): both instantaneous temperature at the time of satellite image acquisition, and daily mean air temperature between the image date and the water-quality sampling date are considered;
4. Wind speed (in m/s): both instantaneous wind speed at the time of satellite image acquisition and the daily mean wind speed between the image date and the water-quality sampling date are considered; and
5. Noon solar altitude (in degrees): the mean noon solar altitude between the image date and the water-quality sampling date.

Instantaneous temperature and wind speed were interpolated from the hourly historical data [54]. And further considering Eqs 4 to 8, the full list of variables considered in the multiple regression process is provided in Table 7. A look-up table between variable abbreviations and variable descriptions is provided as Table 8. As described above, in this study, the band number is based on band-numbering scheme for TM and ETM+.

**Table 7:** Reflectance bands (i.e., band (B1), band 2 (B2), etc.) and ratios used in the variable-selection process.

Water constituent	# of valid observations	Initial predictor variables before p-threshold test
TSS	28	B1, B2, B3, B4, B2/B1, B3/B1, B4/B1, B3/B2, B4/B2, B4/B3, D_off_, T_s_, T_a_, T_mean_, T_s_-T_a_, T_s_-T_mean_, W, W_mean_, Alt, Alt^2^
TN	38	

**Table 8:** Look-up table for variable abbreviation and description of variables.

Variable abbreviations	Variable description
B1, B2, B3, B4	Reflectance value for Band 1, Band 2, Band 3, and Band 4, respectively.
D_off_	Date offset between the image date and the water-quality sampling date
T_s_	Water surface temperature derived from the remote-sensor thermal band
T_a_	Instantaneous air temperature at time of satellite image acquisition
T_mean_	Daily mean air temperature between the image date and the water quality sampling date
W	Instantaneous wind speed at the time of satellite image acquisition
W_mean_	Daily mean wind speed between the image date and the water quality sampling date
Alt	Mean noon solar altitude between the image date and the water-quality sampling date

Even though SWR_cs_ and associated LWR_net_ are not considered in selection of variables, LHF (latent heat flux) and SHF (sensible heat flux) already sum to 2/3 of the upwelling energy budget [69]. Further considering that a few environmental factors (e.g. *T*_*s*_ − *T*_*a*_) also play a role in long-wave radiation LWR_net_, the portion of the upwelling energy flux explained by the environmental factors should be higher than 2/3.

Selection of predictor variables is based on a hybrid forward selection that considers the variation inflation factor (VIF). In conventional forward selection, variables are added to the regression one at a time, starting with no predictor variables being selected. The p-value threshold includes a predictor in the regression equation if its p-value is below a “probability to enter,” and includes a predictor that will most improve the fit first (i.e., “forward”). A default value of 0.25 in JMP [70] was used for “probability to enter”.

In addition to p-value, the variation inflation factor (VIF) was used to minimize multicollinearity of the model. Multicollinearity occurs when a predictor variable is a linear combination of other predictor variables in the model. The direct consequence of multicollinearity is that the error variance is inflated, which may result in low prediction power if the overfitted model is used with a new set of data. VIF is calculated as:

VIFj=1(1−Rj2) (9)

where $R_{j}^{2}$ is the multiple coefficient of determination between the j-th predictor variable of interest and the rest of the predictor variables. The rule of thumb to avoid serious multicollinearity is that all chosen predictor variables should have VIF less than 10 [71]. Unlike other criteria such as Akaike Information Criterion (AIC), Bayesian Information Criterion (BIC), and Mallow’s Cp, VIF is generated for each predictor variable. Also, VIF has a suggested absolute criterion, whereas other criteria (AIC, BIC, Cp, etc.) provide only relative comparison between models.

**Fig 2:**
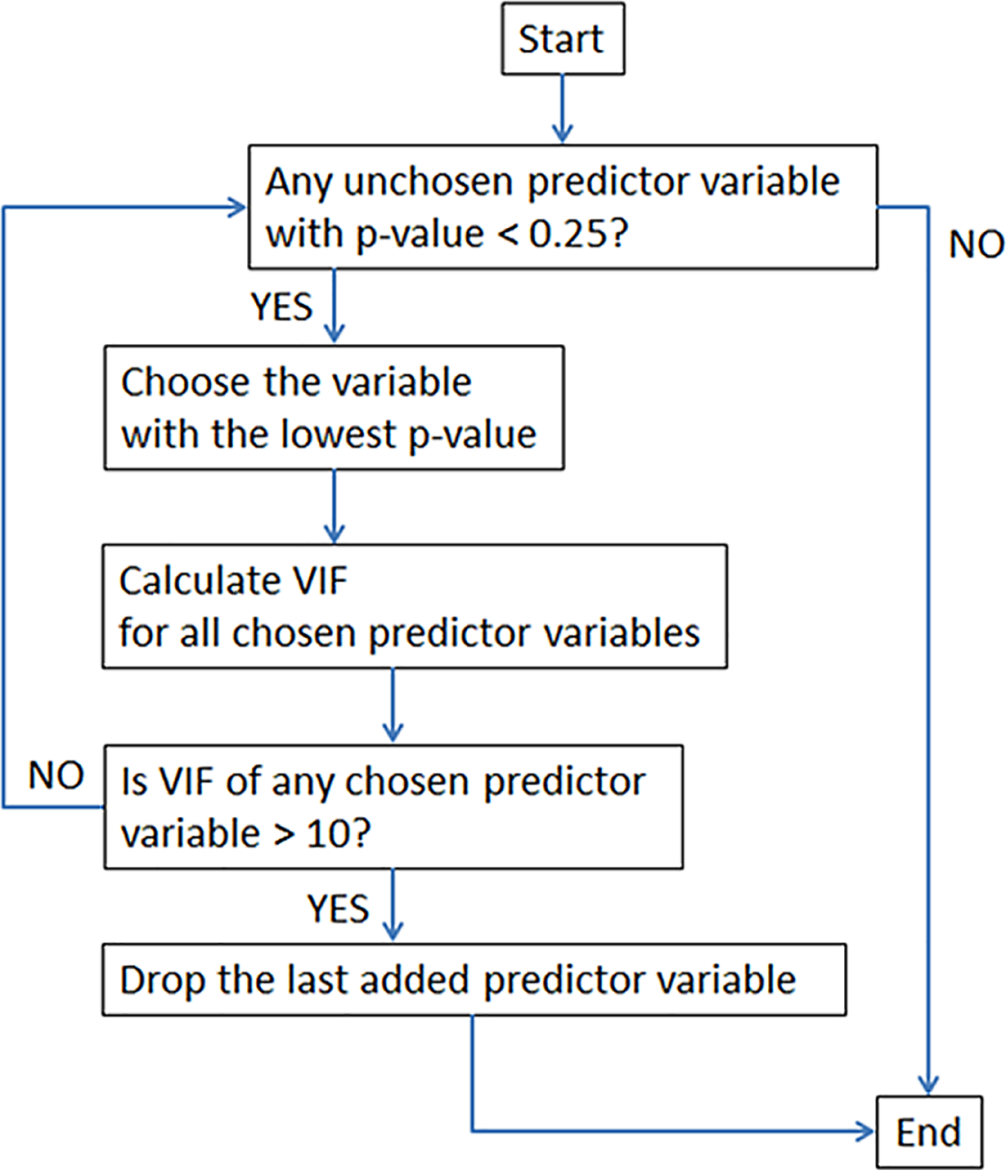
Flow chart of the hybrid forward-selection process for selecting predictor variables in multiple regression analysis.

We use an approach that treats the p-value and VIF equally while adding variables in forward selection. When a variable is added according to the p-value (i.e. conventional rules of forward selection), VIFs of all included variables (including the one that is just added) are also checked. If VIFs are all below the threshold of 10 (or any user-defined value), the newly-added variable is allowed, and the next variable is chosen according to the rule of forward selection. However, if any VIF is found to be larger than the threshold for any of the variables, the most recently-added variable is deleted and the selection procedure stops. Coefficients of variables, p-values, and VIF are dynamically recalculated when any variable is deleted from the model. The procedure is illustrated in Fig 2.

The derived multiple linear equations were then validated by splitting all data into the calibration and validation groups. 80% of the data were used for calibration and the remaining 20% were used for validation because the minimum number of observation samples used in calibration should be approximately twenty [5]. The calibration and validation processes were repeated fifteen times for each of the water-quality constituent using randomly selected calibration and validation data groups. In each of the fifteen runs, the same calibration and validation data groups were used by both the hybrid and conventional forward-selection processes, so their performance can be correctly compared.

## 4. Results

The statistics of coefficients of determination from all fifteen calibration and validation runs were provided in Tables 9 and 10. The results showed good prediction accuracy for future TSS data, but less than satisfactory validation accuracy for TN [72]. Compared to conventional forward selection, validation runs have higher accuracy for both TSS and TN with the utilization of the hybrid forward-selection process. More discussions based on Tables 9 and 10 will be provided later in this paper.

**Table 9:** Calibration and validation results for TSS.

	Hybrid Forward Selection		Conventional Forward Selection	
	Calibration	Validation	Calibration	Validation
**Mean R**^**2**^	0.73	0.70	0.76	0.63
**Standard deviation of R**^**2**^	0.06	0.11	0.06	0.21

**Table 10:** Calibration and validation results for TN.

	Hybrid Forward Selection		Conventional Forward Selection	
	Calibration	Validation	Calibration	Validation
**Mean R**^**2**^	0.64	0.37	0.76	0.33
**Standard deviation of R**^**2**^	0.07	0.21	0.10	0.21

After showing the proposed procedure can provide adequate calibration accuracy and improved validation accuracy compared to the conventional approach, a set of “best” predictive equations using all available data was created and provided in Table 11 for use in the subsequent discussions and field applications. The results in Table 11 include the predictor variables, importance of the predictor variable, associated regression coefficients and standard error, 95% confidence intervals for the regression coefficients, p-values, and VIF values for each of the response variables (TSS and TN). The importance values (“Imp. of Var.”) are calculated by dividing the change in R^2^ (coefficient of determination) when the variable of interest is dropped from the model by the overall R^2^ when the variable of interest is included [73]. The sum of importance values of all variables does not equal to 1 since the importance is relative.

**Table 11:** Best fitting multiple regression models for TSS and TN using the hybrid forward selection considering VIF.

					Coefficient of predictor		Confidence Interval for coefficient			
ResponseVariable	R^2^	Num. of Obs.	Pred. Variable	Imp. of Var.	Value	Std. Error	Lower 95%	Upper 95%	p	VIF
$\sqrt[{2}]{TSS}$	0.68	28	(intercept)	-	-0.67	0.50	-1.69	0.36	0.19	-
			B3/B1	0.93	1.67	0.24	1.16	2.17	<0.0001	1.21
			W	0.21	0.21	0.065	0.077	0.34	0.0034	1.08
			T_s_-T_mean_	0.04	0.038	0.027	-0.018	0.093	0.18	1.16
$\sqrt[{2}]{TN}$	0.62	38	(intercept)	-	4.357	0.91	2.50	6.21	<0.0001	-
			W_mean_	0.39	-0.0533	0.012	-0.078	-0.029	<0.0001	1.26
			T_s_	0.32	-0.0124	0.0031	-0.019	-0.0062	0.0003	1.31
			B1	0.18	4.497	1.50	1.44	7.55	0.0053	2.35
			B4/B1	0.11	-0.0493	0.020	-0.090	-0.0089	0.018	2.73
			D_off_	0.11	-0.0126	0.0051	-0.023	-0.0021	0.020	1.18
			B2/B1	0.05	0.106	0.067	-0.030	0.24	0.12	4.50

The resulting best multiple regression-based models are provided in Eqs 10 and 11:

$$TSS=(-0.67+1.67\times \frac{B3}{B1}+0.21\times W+0.038\times (T_{s}-T_{mean}){)}^{2}$$ (10)

$$TN=(4.357-0.0533\times W_{mean}-0.0124\times T_{s}+4.497\times B1-0.0493\times \frac{B4}{B1}-0.0126\times D_{off}+0.106\times \frac{B2}{B1}{)}^{2}$$ (11)

**Fig 3:**
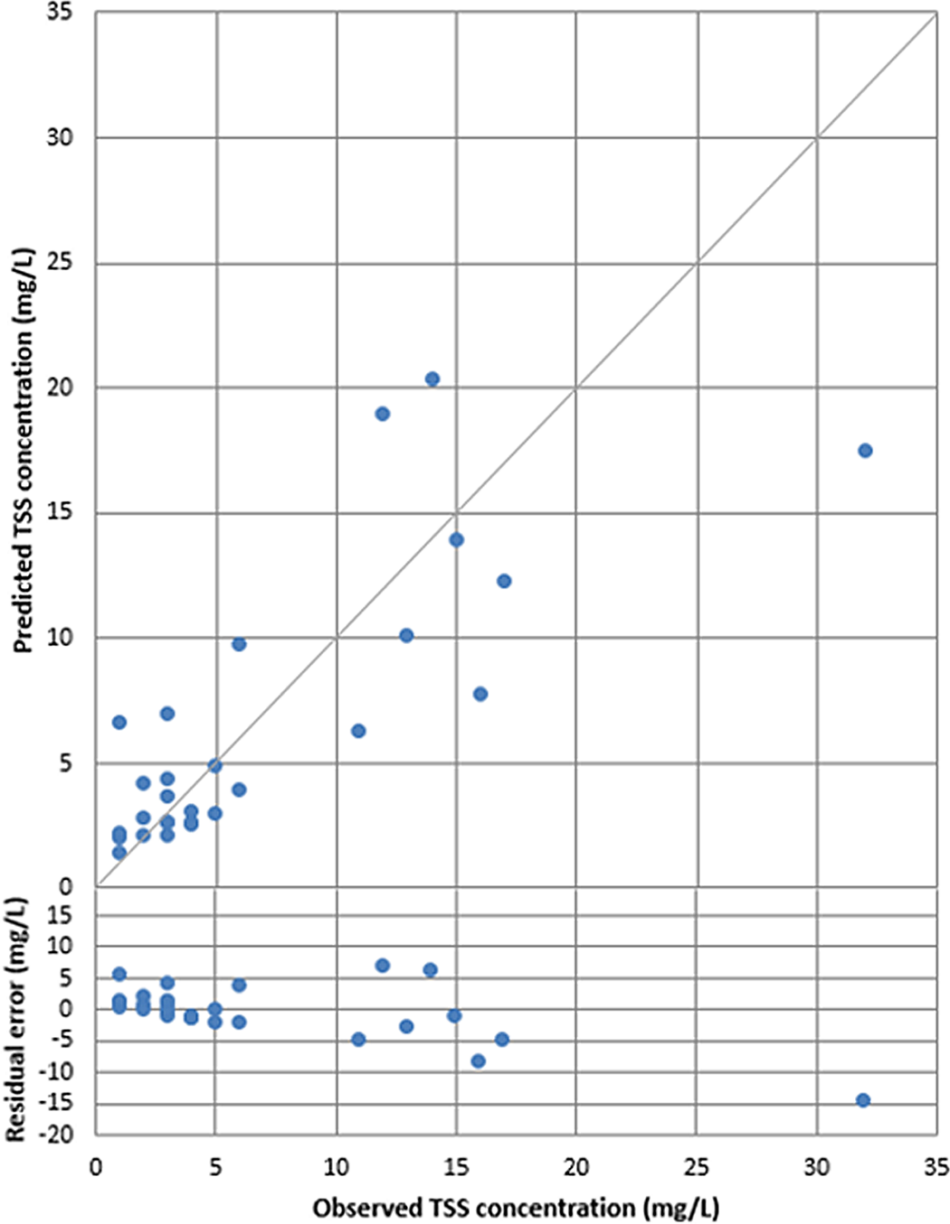
Observed versus predicted values for total suspended solids (TSS) (R^2^ = 0.68).

**Fig 4:**
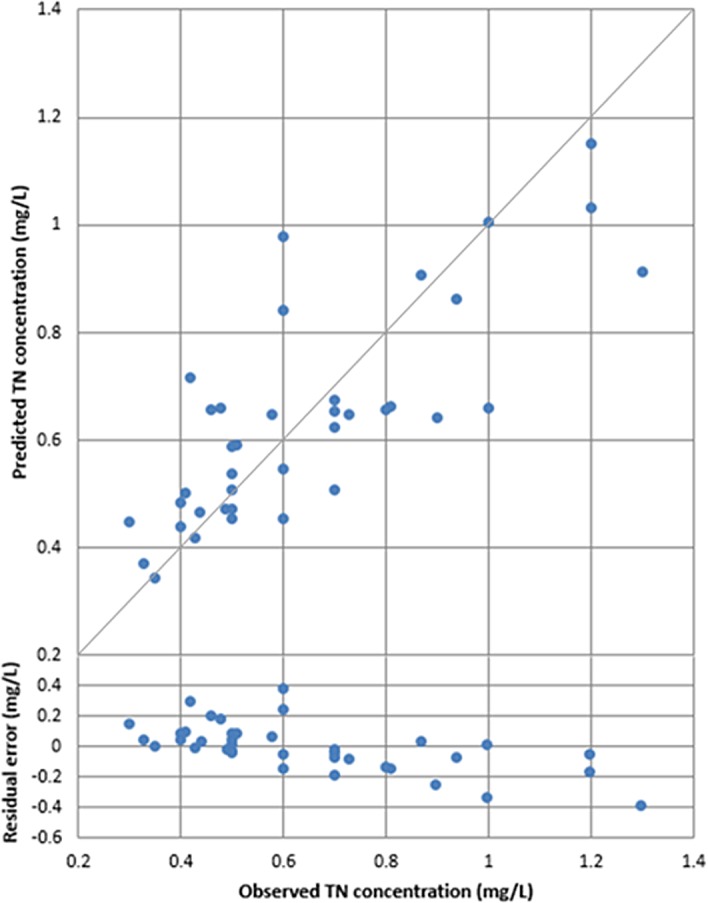
Observed versus predicted values for total nitrogen (TN) (R^2^ = 0.62).

Plots of the observed versus predicted concentrations for the best predictive equations (based on all available data) of TSS and TN calculated from Eqs 10 and 11 are plotted in Figs 3 and 4 respectively. The residual error (defined as the predicted value minus the observed value) and 1:1 line is added to both figures.

## 5. Discussion

The multiple linear equations derived from the regression analysis indicate that weather-related variables play an important role in predicting water-quality measures. In fact, many weather variables bear more importance than the multispectral variables do. The relative importance of each variable is provided in Table 11. If all the weather variables are removed from Table 11, the predictive variables related to Landsat bands alone provide only coefficients of determination, R^2^, of 0.53 and 0.26 for TSS and TN respectively.

Given the statistics of the fifteen calibration and validation runs, prediction of TSS concentration is accurate, with the hybrid process providing improved accuracy. Even though the calibration accuracy for TN is satisfactory, the validation accuracy is not, and the standard deviation of validation R^2^ is relatively high. However, we observed that the derived equations can still predict the relative magnitude of TN concentrations. Therefore, we suggest using the TN equation to evaluate the trend of change in water quality only.

The VIF-based hybrid forward-selection process showed better performance than that of the conventional forward selection process. In some of the runs, the hybrid process and the conventional process arrived at the same equations, but the hybrid process successfully prevented overfitting in other runs, thus resulting in higher validation accuracy. Due to overfitting, calibration accuracy from the conventional process is higher than that of the hybrid process, with the cost of lower validation accuracy.

Kloiber et al. [12] found that both B1 and the ratio B3/B1 can be used to predict the Secchi disk transparency, which is closely related to TSS. From Kloiber et al. [12], the regression model containing B3/B1 and B1 predicted Secchi disk transparency with R^2^ of 0.75. We also found B3/B1 as the dominant important variable in determining TSS concentrations, but did not find B1 as one of the significant prediction variables. Kloiber et al. [12] accrued a slightly higher R^2^ than our study possibly because Kloiber et al. limited their *in situ* data collection to ±1 day from the corresponding satellite image acquisitions. In the current study, the predictive equation that includes B3/B1 alone has a R^2^ of 0.53 for TSS because our available data only allows *in situ* samples to be ±7 days from satellite image acquisitions. Considering weather variables successfully boosted R^2^ to 0.68, such that it was comparable with that of Kloiber et al. [12] (i.e., 0.75).

For TSS, we found the instantaneous wind speed, W, to be an important prediction variable. Since the instantaneous wind speed is chosen, instead of the daily mean wind speed between the image date and the water-quality sampling date (W_mean_), it indicates that the instantaneous effect of wind (such as the surface ripple effect) is more important to TSS determination than the long-term heat-exchange effect. Even though the difference between the water surface temperature and the daily mean air temperature between the image date and the water-quality sampling date is selected as one of the prediction variables, it is of little importance in the model. It was chosen because the default forward-selection method has a lenient inclusion criterion (p = 0.25).

Dewidar and Khedr [11] determined that the band ratio B2/B1 is important in determining the TN concentration in brackish lagoons. However, the correlation between B2/B1 and TN was low in Dewindar and Khedr [11], with a correlation coefficient of 0.298. B2/B1 was also chosen by this study as one of the predictor variables, but B2/B1 still bears little predictive power as shown in Table 11. In contrast, the daily mean wind speed between the image date and the water-quality sampling date (W_mean_) and water surface temperature (T_s_) were determined to be the two most important predictor variables for TN prediction.

The high importance of water surface temperature T_s_ fortifies the hypothesis that water temperature is related to the growth of microorganisms. The high importance of the daily mean wind speed between the image date and the water-quality sampling date (W_mean_) and date difference (D_off_) indicate that temperature change due to accumulated heat flux between the image date and sampling date is important. Referring to Eqs 7 and 8, the mechanism involved should be the latent heat flux because latent heat flux (Eq 7) and sensible heat flux (Eq 8) are the only two components in the heat flux budget that involve wind speed. Latent heat flux is a main component of heat exchange between water and the atmosphere, and sensible heat plays a much lesser role [74]. Even though the circumstantial evidence based on Eqs 7 and 8 points to the conclusion noted above, this still needs to be validated by direct evidence from future field experiments.

## 6. Field application

**Fig 5:**
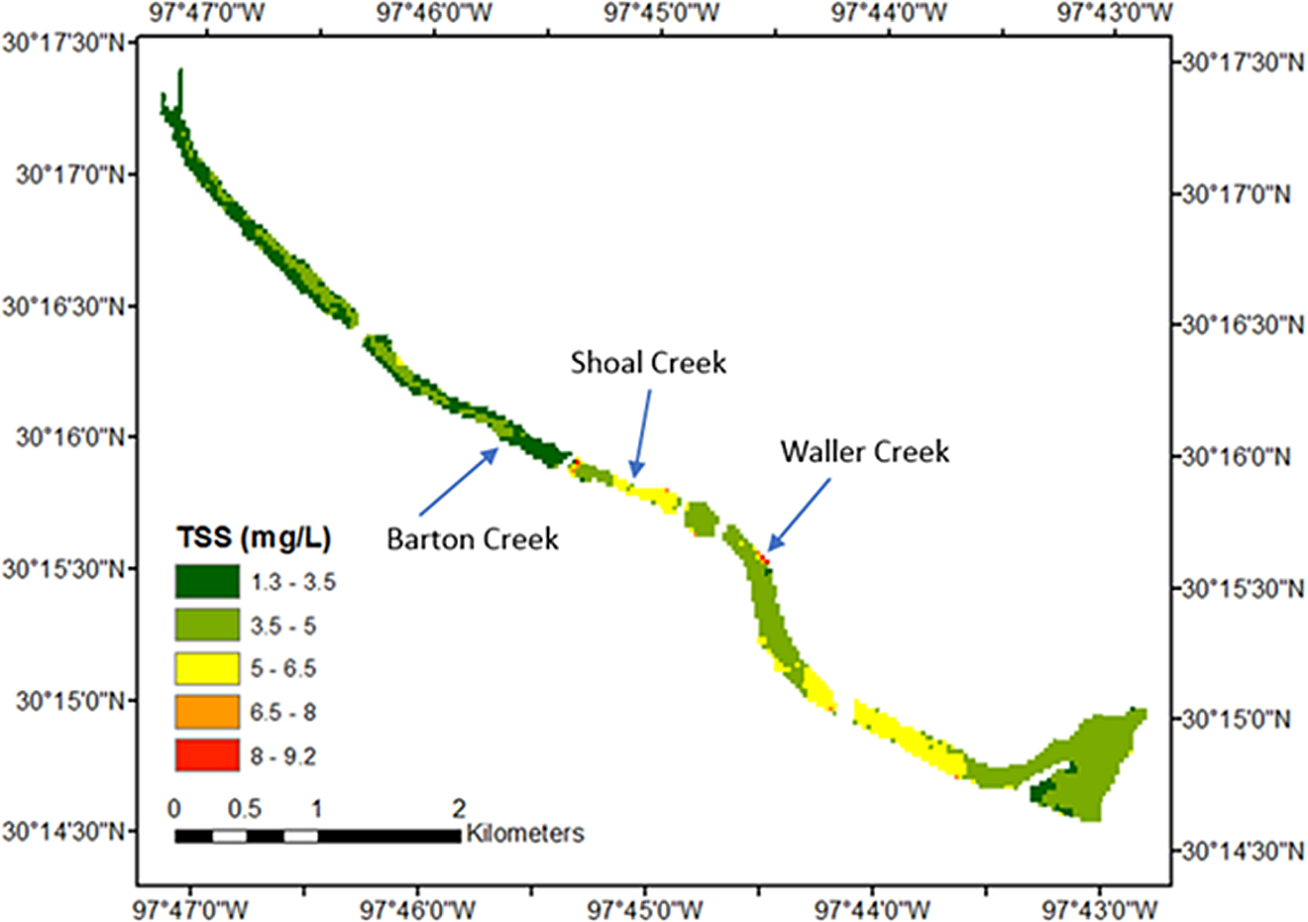
TSS concentrations for Lady Bird Lake, Austin, Texas, USA, May 14, 2014.

**Fig 6:**
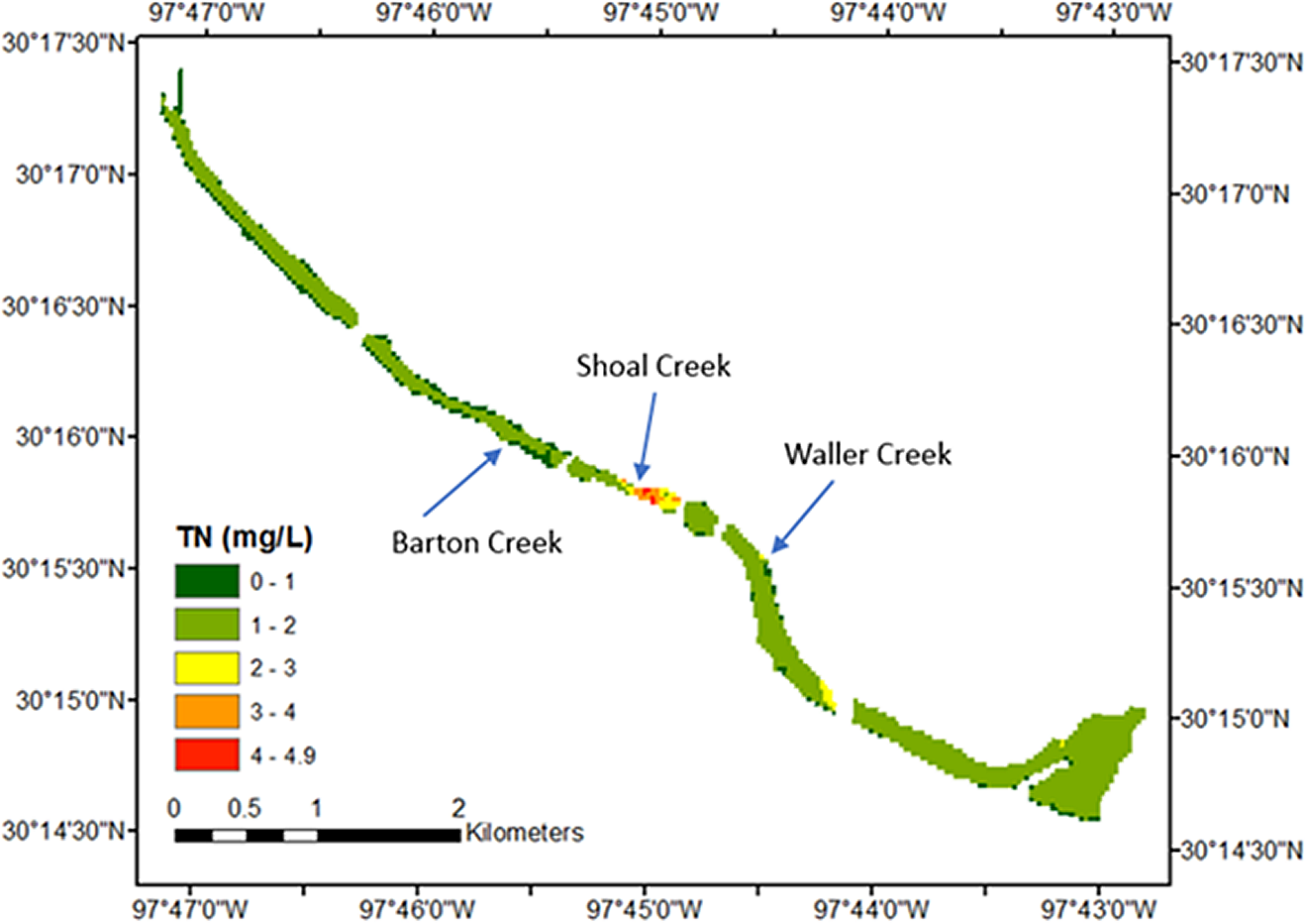
TN concentrations for Lady Bird Lake, Austin, Texas, USA, May 14, 2014.

To demonstrate the utility of water-quality monitoring by satellites via our proposed method, water-quality measures from Lady Bird Lake on May 14, 2014 were estimated using Eqs 10 and 11, respectively. This date was chosen because storms occurred on the day previous to and in the morning of the satellite overpass (prior to the overpass) with a cumulative rainfall depth of 27 mm, likely making it easier to discern the effect of urban stormwater runoff to the lake. Figs 5 and 6 give the respective predicted spatial distribution of TSS and TN concentrations.

The water quality in the northwestern part of the lake is generally better than that in the southeastern area, which is expected as a result of urban runoff. Lady Bird Lake has three major tributaries in the metropolitan Austin area: Barton Creek, Shoal Creek, and Waller Creek. The confluence points of the three streams are indicated in Figs 5 and 6. Barton Creek includes an extensive green belt around its riparian zone, and strict development regulations are in force because it is located within the Edwards Aquifer recharge zone [75]. As a result, there is no marked change in TSS and TN at the confluence point of Barton Creek, relative to proximal areas of the lake. However, the confluence points of Shoal Creek and Waller Creek show significant increase in TSS and TN. This illustrates the effect of conservation efforts spent on each watershed on water quality. The influence of Shoal Creek is more visible in Figs 5 and 6 than that of Waller Creek because Shoal Creek has a larger drainage area [76]. Such details in spatial distribution can only be achieved via satellite-derived water-quality predictions and can serve as the precursor examination for more detailed water-quality examinations. This field application also showed that the equation to predict TN concentration can estimate the trend of change in TN concentration with sufficient confidence, even though the predicted absolute concentration values might not be accurate.

## 7. Conclusions

Multiple regression-derived equations using reflectance bands, band ratios, and environmental factors as predictor variables for concentrations of TSS and TN respectively, were derived using a hybrid forward-selection method that considers both VIF and p-value in the forward-selection process. Landsat TM, ETM+, and OLI/TIRS (Landsat 8) images were all used to derive the single set of equations. The coefficients of determination of the best-fitting resultant equations are 0.68 for TSS and 0.62 for TN based on all available observation data. Through repeated data splitting into the calibration and validation groups, the hybrid method delivered a calibration accuracy (in R^2^) of 0.73 and 0.64 for TSS and TN, respectively, and validation accuracy of 0.70 and 0.37 for TSS and TN, respectively. The hybrid forward-selection process consistently showed better validation accuracy compared to that of the conventional forward-selection process. Validation results show good accuracy for TSS prediction. However, the mean and standard deviation for coefficients of determination of TN validations tends to be unsatisfactory. Therefore, the predictive equation for TN is recommended for trend evaluation only, as indicated by the field application.

Among all chosen predictor variables, B3/B1 has the strongest influence on the predictive power for TSS retrieval. The band ratio of B3/B1 was also selected by Kloiber et al. [12] in predicting Secchi disc transparency, indicating a correlation between Secchi disc transparency and TSS. Other reflectance bands and band ratios, such as B1, B2/B1, and B4/B1 are also influential in estimating TN concentrations, but they are not dominant factors.

Environmental factors, such as wind speed and water surface temperature, were crucial in determination of water quality in this study. Inclusion of environmental factors allows usage of a single set of predictive equations across the seasons, as such predictive equations are innately adapted to the environmental changes for different seasons. The predictive equation will also likely to be more accurate because the pooling of all observation data.

The instantaneous wind speed, W, bears considerable importance in TSS determination, which is explained by wind-generated surface ripple effects. Water surface temperature T_s_ (derived from satellite remote-sensor thermal band image data) is important in determination of TN concentrations, as the growth of microorganisms in water is correlated with water nutrient concentrations.

The time offset between the satellite image-acquisition date and water-sampling date must be accounted for in water nutrient concentration (i.e., TN) retrieval. The heat flux budget between air and the water surface was considered, and components in the budget equations were included in the forward-selection procedure. In additional to the predictor variables identified above, the daily mean wind speed between the image-acquisition date and water-sampling date (W_mean_) was identified as the most important predictor variables for TN determinations. The time difference (in days) between the image-acquisition date and water-sampling date (D_off_) was also chosen for TN determination. According to the heat flux budget equations, the inclusion of W_mean_, T_s_, and D_off_ indicates the dominance of latent heat flux in the determination of TN.

The results showed that:

1. Environmental factors can constitute important ancillary variables in water quality estimation based on satellite remote-sensor images;
2. By including environmental factors, it is feasible to pool all observation data to create a single set of predictive equations, and use it to estimate water quality for all seasons;
3. A single set of predictive equations can be determined to retrieve year-round water-quality quantities (e.g., TSS and TN) with satisfactory accuracy from Landsat TM, ETM+, and OLI/TIRS imagery on the same lacustrine water body;
4. Population change does not drastically change the applicability (i.e. the relationship between spectral bands and water-quality constituents) of regression-derived equations for water quality prediction. The derived predictive equations are applicable for data across 30+ years (1983 to 2015) even though population in the metropolitan area almost tripled (from 374,000 in 1983 to 901,000 in 2015) over the same period of time [44];
5. Including VIF as part of the forward-selection process comprises a simple yet reliable methodology for choosing predictor variables for TSS concentrations;
6. Prediction of water nutrient concentrations (e.g., TN) yields low accuracy using the methodology depicted in this study, but the predictive equations are still valuable in evaluating the trends of spatial and temporal changes of nutrient concentrations; and
7. The methodology depicted in this study, including the utilization of a hybrid forward-selection process and consideration of environmental factors, showed marked improvements compared to the conventional methods, and it is simple enough to be followed by government agencies by addressing the issues of cost, product accuracy, data continuity, and programmatic support.

In the future, the hybrid forward-selection method can be further refined to require a stricter criterion for the inclusion of predictor variables. The default p = 0.25 may have allowed inclusion of a few predictor variables that were not significant in the final selection of variables.

In addition, inclusion of ancillary environmental factors involving long-term averaging, such as average wind speed (W_mean_), into the regression models demonstrated that it is possible to satisfactorily estimate water-quality quantities, even when a large temporal offset between satellite image-acquisition and *in situ* water sampling exists. Currently, the recommended longest temporal window between remote-sensor image-acquisition and water-sampling date is approximately seven days [20]. Since these environmental factors are part of the heat flux equations, including environmental factors in predictive equations means an active compensation in estimation error due to the temporal offset in collecting image and water-sample data. This hypothesis needs further testing as part of future research efforts.

## References

[1] Kannel PR., Lee S, Kanel SR, Khan SP, Lee Y-S. Spatial-temporal variation and comparative assessment of water qualities of urban river system: a case study of the river Bagmati (Nepal). Environ. Monit. Assess. 2007; 129: 433–459. 10.1007/s10661-006-9375-6 17242978

[2] Harmel RD, King KW, Haggard BE, Wren DG, Sheridan JM. Practical guidance for discharge and water quality data collection on small watersheds. Tran. ASABE 2006; 49(4): 937–948.

[3] United States Geological Survey. USGS water data for the nation. Available from: http://waterdata.usgs.gov/nwis Cited 5 January 2018.

[4] McCullough IM, Loftin CS, Sader SA. High-frequency remote monitoring of large lakes with MODIS 500 m imagery. Remote Sens. Environ. 2012; 124: 234–241.

[5] Liu Y, Islam MA, Gao J. Quantification of shallow water quality parameters by means of remote sensing. Prog. Phys. Geog. 2003; 27(1): 24–43.

[6] Bukata RP. Satellite monitoring of inland and coastal water quality: Retrospection, introspection, future directions Boca Raton, FL, USA: Taylor and Francis Group; 2005.

[7] Song K, Liu D, Li L, Wang Z, Wang Y, Jiang G. Spectral absorption properties of colored dissolved organic matter (CDOM) and total suspended matter (TSM) of inland waters Goldberg MD and Bloom HJ (Eds), Proc. of SPIE Vol. 7811, 78110B, SPIE 2010; 1–13.

[8] Gitelson AA. The peak near 700 nm on radiance spectra of algae and water: relationships of its magnitude and position with chlorophyll concentration. Int. J. of Remote Sensing 1992; 13: 3367–3373.

[9] Doxaran D, Froidefond J-M, Lavender S, Castaing P. Spectral signature of highly turbid waters application with SPOT data to quantify suspended particulate matter concentrations. Remote Sens. Environ. 2002; 81: 149–161.

[10] Wu G, Leeuw JD, Liu Y. Understanding seasonal water clarity dynamics of Lake Dahuchi from in situ and remote sensing data. Water Reso. Manage. 2009; 23: 1849–1861.

[11] Dewidar KH, Khedr A. Water quality assessment with simultaneous Landsat-5 TM at Manzala Lagoon, Egypt. Hydrobiologia. 2001; 457: 49–58.

[12] Kloiber SM, Brezonik PL, Olmanson LG, Bauer ME. A procedure for regional lake water clarity assessment using Landsat multispectral data. Remote Sens. Environ. 2002; 82: 38–47.

[13] Kishino M, Tanaka A, Ishizaka J. Retrieval of Chlorophyll a, suspended solids, and colored dissolved organic matter in Tokyo Bay using ASTER data. Remote Sens. Environ. 2005; 99: 66–74.

[14] Filippi AM. Derivative-neural spectroscopy for hyperspectral bathymetric inversion. Prof. Geogr. 2007; 59(2): 236–255.

[15] Schaeffer BA, Schaeffer KG, Keith D, Lunetta RS, Conmy R, Gould RW. Barriers to adopting satellite remote sensing for water quality management. Int. J. Remote Sens. 2013; 34(21): 7534–7544.

[16] National Aeronautics and Space Administration. Free Landsat 7 data available from USGS. Available from: http://landsat.gsfc.nasa.gov/free-landsat-7-data-available-from-usgs/ Cited 6 January 2018.

[17] National Aeronautics and Space Administration. About MODIS: Moderate resolution imaging spectroradiometer. Available from: https://modis.gsfc.nasa.gov/about/ Cited 6 January 2018.

[18] National Aeronautics and Space Administration. SeaWiFS project. Available from: https://oceancolor.gsfc.nasa.gov/SeaWiFS/ Cited 6 January 2018.

[19] United States Geological Survey. Predicting water quality by relating Secchi-disk transparency and Chlorophyll a measurements to satellite imagery for Michigan inland lakes, August 2002 Reston, VA, USA: U.S. Geological Survey; 2004.

[20] Barrett DC, Frazier AE. Automated method for monitoring water quality using Landsat imagery. Water. 2016; 8: 257.

[21] Kallio K, Attila J, Harma P, Koponen S, Pulliainen J, Hyytiainen U-M, Pyhalahti T. Landsat ETM+ images in the estimation of seasonal lake water quality in boreal river basins. Environ. Manage. 2008; 42: 511–522. 10.1007/s00267-008-9146-y 18509700

[22] Okin GS, Gu J. The impact of atmospheric conditions and instrument noise on atmospheric correction and spectral mixture analysis of multispectral imagery. Remote Sens. Environ. 2015; 164: 130–141.

[23] Karakaya N, Evrendilek F. Monitoring and validating spatio-temporal dynamics of biogeochemical properties in Mersin Bay (Turkey) using Landsat ETM+. Environ. Monit. Assess. 2011; 181: 457–464. 10.1007/s10661-010-1841-5 21181257

[24] Lim J, Choi M. Assessment of water quality based on Landsat 8 operational land imager associated with human activities in Korea. Environ. Monit. Assess. 2015; 187: 384 10.1007/s10661-015-4616-1 26017808

[25] Rasconi S, Gall A, Winter K, Kainz MJ. Increasing water temperature triggers dominance of small freshwater plankton. PLOS ONE. 2015; 10(10): e0140449 10.1371/journal.pone.0140449 26461029 PMC4603799

[26] Voutilainen A, Jurvelius J, Lilja J, Viljanen M, Rahkola-Sorsa M. Associating spatial patterns of zooplankton abundance with water temperature, depth, planktivorous fish and chlorophyll. Boreal Environ. Res. 2015; 21: 101–114.

[27] Bonansea M, Rodriguez MC, Pinotti L, Ferrero S. Using multi-temporal Landsat imagery and linear mixed models for assessing water quality parameters in Río Tercero reservoir (Argentina). Remote Sens. Environ. 2015; 158: 28–41.

[28] Alparslan E, Coskun HG, Alganci U. An investigation on water quality of Darlik Dam drinking water using satellite images. Thescientificworldj. 2010; 10: 1293–1306. 10.1100/tsw.2010.125 PMC5763954 20623088

[29] Wang Y, Xia H, Fu J, Sheng G. Water quality change in reservoirs of Shenzhen, China: detection using LANDSAT/TM data. Sci. Total Environ. 2004; 328: 195–206. 10.1016/j.scitotenv.2004.02.020 15207584

[30] Wu G, Leeuw JD, Skidmore AK, Prins HHT, Liu Y. Comparison of MODIS and Landsat TM5 images for mapping tempo-spatial dynamics of Secchi disk depths in Poyang Lake National Nature Reserve, China. Int. J. Remote Sens. 2008; 29(8): 2183–2198.

[31] Zhou Z, Wang X. Quantitative Remote Sensing Researches on Water Quality of the Weihe River Based on SPOT-5 Imagery. Proceedings of Information Technology and Environmental System Sciences. 2008: 538–542.

[32] Lovric M. International encyclopedia of statistical science Berlin Heidelberg, Germany: Springer-Verlag; 2011.

[33] Gilmour SG. The interpretation of Mallow’s Cp-statistic. J. R. Stat. Soc. 1996; 45(1): 49–56.

[34] Hair JF Jr., Black WC, Babin BJ, Anderson RE. Multivariate Data Analysis A Global Perspective 7th ed Upper Saddle River, NJ, USA: Pearson; 1998.

[35] Graham MH. Confronting multicollinearity in ecological multiple regression. Ecology. 2003; 84(11): 2809–2815.

[36] Yang J, Weisberg PJ, Bristow NA. Landsat remote sensing approaches for monitoring long-term tree cover dynamics in semi-arid woodlands: Comparison of vegetation indices and spectral mixture analysis. Remote Sens. Environ. 2012; 119: 62–71.

[37] Alsharif AAA, Pradhan B. Urban sprawl analysis of Tripoli Metropolitan City (Libya) using remote sensing data and multivariate logistic regression model. J. Indian Soc. Remote Sens. 2014; 42(1): 149–163.

[38] Dubovyk O, Menz G, Conrad C, Kan E, Machwitz M, Khamzina A. Spatio-temporal analyses of cropland degradation in the irrigated lowlands of Uzbekistan using remote-sensing and logistic regression modelling. Environ Monit Assess. 2012; 185: 4775–4790. 10.1007/s10661-012-2904-6 23054271 PMC3641299

[39] Sun D, Hu C, Qiu Z, Shi K. Estimating phycocyanin pigment concentration in productive inland waters using Landsat measurements: A case study in Lake Dianchi. Opt Express. 2015; 23(3): 3055–3074. 10.1364/OE.23.003055 25836166

[40] Zheng Z, Li Y, Guo Y, Xu Y, Liu G, Du C. Landsat-based long-term monitoring of total suspended matter concentration pattern change in the wet season for Dongting Lake, China. Remote Sens. 2015; 7: 13975–13999.

[41] Lymburner L, Botha E, Hestir E, Anstee J, Sagar S, Dekker A, Malthus T. Landsat 8: Providing continuity and increased precision for measuring multi-decadal time series of total suspended matter. Remote Sens. Environ. 2016; 185: 108–118.

[42] Mishra N, Haque MO, Leigh L, Aaron D, Helder D, Markham B. Radiometric cross calibration of Landsat 8 Operational Land Imager (OLI) and Landsat 7 Enhanced Thematic Mapper Plus (ETM+). Remote Sens. 2014; 6: 12619–12638.

[43] United States Geological Survey. Landsat missions. Available from: http://landsat.usgs.gov/index.php Cited 6 January 2018.

[44] City of Austin. Demographic data. Available from: http://www.austintexas.gov/page/demographic-data Cited 20 June 2018.

[45] City of Austin. What are some general characteristics of each lake? Available from: https://www.austintexas.gov/faq/what-are-some-general-characteristics-each-lake Cited 6 January 2018.

[46] Texas Water Development Board. Volumetric Survey of Lady Bird Lake. Austin, TX, USA: Texas Water Development Board; 2009.

[47] United Stated Geological Survey. USGS global visualization viewer. Available from: http://glovis.usgs.gov/ Cited 6 January 2018.

[48] City of Austin. 2014 Land use inventory. Available from: ftp://ftp.ci.austin.tx.us/GIS-Data/planning/maps/Landuse%20Inventory%202014.pdf Cited 6 January 2018.

[49] United States Department of Agriculture. Urban hydrology for small watersheds (TR-55). Available from: https://www.nrcs.usda.gov/Internet/FSE_DOCUMENTS/stelprdb1044171.pdf Cited 6 January 2018.

[50] Perkins T, Adler-Golden S, Matthew M, Berk A, Anderson G, Gardner J. Retrieval of atmospheric properties from hyper- and multi-spectral imagery with the FLAASH atmospheric correction algorithm. P. Soc. Photo-Opt. Ins. 2005; 5979: 1–11.

[51] Exelis, Inc. Atmospheric Correction Module: QUAC and FLAASH User’s Guide. ver. 4.7. Boulder, CO, USA: Exelis Inc.; 2009.

[52] Siegel DA, Wang M, Maritorena S, Robinson W. Atmospheric correction of satellite ocean color imagery: the black pixel assumption. Appl. Optics 2000; 39(21): 3582–3591. 10.1364/ao.39.003582 18349929

[53] Hadjimitsis DG, Clayton CR, Hope VS. An assessment of the effectiveness of atmospheric correction algorithms through the remote sensing of some reservoirs. Int. J. Remote Sens. 2004; 25(18): 3651–3674.

[54] National Oceanic and Atmospheric Administration. National weather service automated surface observing system. Available from: http://www.nws.noaa.gov/asos/ Cited 6 January 2018.

[55] National Oceanic and Atmospheric Administration. TAF Decoder. Available from: https://www.aviationweather.gov/static/help/taf-decode.php Cited 23 May 2018.

[56] National Aeronautics and Space Administration. MODIS total precipitable water. Available from: https://modis.gsfc.nasa.gov/data/dataprod/mod05.php Cited 6 January 2018.

[57] Barsi JA, Schott JR, Palluconi FD, Hook SJ. Validation of a web-based atmospheric correction tool for single thermal band instruments. Proceedings of SPIE. 2005: 58820E1–58820E7.

[58] Hayden C. M. 1988 GOES-VAS simultaneous temperature moisture retrieval algorithm. J. Appl. Meteor. 27: 705–733.

[59] Lamaro A.A., Marinelarena A., Torrusio S.E., and Sala S.E. 2013 Water surface temperature estimation from Landsat 7 ETM+ thermal infrared data using the generalized single-channel method: Case study of Embalse del Río Tercero (Córdoba, Argentina). Advances in Space Research. 51(3): 492–500.

[60] Barsi JA, Schott JR, Hook SJ, Raqueno NG, Markham BL, Radocinski RG. Landsat-8 thermal infrared sensor (TIRS) vicarious radiometric calibration. Remote Sens. 2014; 6: 11607–11626.

[61] Frazier PS, Page KJ. Water body detection and delineation with Landsat TM data. Photogramm. Eng. Rem. S. 2000; 66(12): 1461–1467.

[62] Jensen JR. Remote sensing of the environment—an earth resource perspective 2nd ed Upper Saddle River, NJ, USA: Prentice Hall; 2007.

[63] Boyer JN, Kelble CR, Ortner PB, Rudnick DT. Phytoplankton bloom status: Chlorophyll a biomass as an indicator of water quality condition in the southern estuaries of Florida, USA. Ecol. Indic. 2009; 9(6): S56–S67.

[64] Kumar BP, Vialard J, Lengaigne M, Murty VSN, McPhaden MJ. TropFlux: Air-sea fluxes for the global tropical oceans—description and evaluation. Clim. Dynam. 2012; 38(7): 1521–1543.

[65] DeCosmo J, Katsaros KB, Smith SD, Anderson RJ, Oost WA, Bumke K, Chadwick H. Air-sea exchange of water vapor and sensible heat: the Humidity Exchange Over the Sea (HEXOS) results. J. Geophys. Res. 1996; 101: 12001–12016.

[66] Thomson GW. The Antoine equation for vapor-pressure data. Chem. Rev. 1946; 38(1): 1–39. 21016992 10.1021/cr60119a001

[67] Stull RB. Meteorology for scientists and engineers. 3rd ed. Boston, MA, USA: Brooks Cole; 2011.

[68] Gubler S, Gruber S, Purves RS. Uncertainties of parameterized surface downward clear-sky shortwave and all-sky longwave radiation. Atmos. Chem. Phys. 2012; 12: 5077–5098.

[69] NASA Goddard Space Flight Center. Surface energy budget. Available from: https://earthobservatory.nasa.gov/Features/EnergyBalance/page5.php Cited 24 May 2018.

[70] SAS Institute. JMP documentation. Available from: http://www.jmp.com/en_us/support/jmp-documentation.html Cited 6 January 2018.

[71] Chatterjee S, Simonoff JS. Handbook of regression analysis. Somerset, NJ, USA: Wiley Publication; 2013.

[72] Moriasi DN, Arnold JG, Van Liew MW, Bingner RL, Harmel RD, Veith TL. Model evaluation guidelines for systematic quantification of accuracy in watershed simulations. T. ASABE. 2007; 50(3): 885–900.

[73] Judd CM, McClelland GH, Ryan CS. Data analysis: A model comparison approach. New York, NY, USA: Routledge; 2008.

[74] Foltz GR, McPhaden MJ. Mixed layer heat balance on intraseasonal time scales in the northwestern tropical Atlantic Ocean. J. Clim. 2005; 18: 4168–4184.

[75] Ernst C, Gullick R, Nixon K. Conserving forest to protect water. Opflow. 2004; 30(5): 1, 4–7.

[76] City of Austin. City of Austin watershed regulation areas. Available from: http://www.austintexas.gov/sites/default/files/files/Watershed/watershed_regs_map.pdf Cited 6 January 2018.

